# Comparison of analyses of the QTLMAS XIII common dataset. I: genomic selection

**DOI:** 10.1186/1753-6561-4-s1-s1

**Published:** 2010-03-31

**Authors:** John W M Bastiaansen, Marco C A M Bink, Albart Coster, Chris Maliepaard, Mario P L Calus

**Affiliations:** 1 Animal Breeding and Genomics Centre, Wageningen University, Wageningen, The Netherlands; 2 Biometris, Plant Research International, Wageningen, The Netherlands; 3 Plant Breeding, Wageningen University, Wageningen, The Netherlands; 4 Animal Breeding and Genomics Centre, Wageningen UR Livestock Research, Lelystad, The Netherlands

## Abstract

**Background:**

Genomic selection, the use of markers across the whole genome, receives increasing amounts of attention and is having more and more impact on breeding programs. Development of statistical and computational methods to estimate breeding values based on markers is a very active area of research. A simulated dataset was analyzed by participants of the QTLMAS XIII workshop, allowing a comparison of the ability of different methods to estimate genomic breeding values.

**Methods:**

A best case scenario was analyzed by the organizers where QTL genotypes were known. Participants submitted estimated breeding values for 1000 unphenotyped individuals together with a description of the applied method(s). The submitted breeding values were evaluated for correlation with the simulated values (accuracy), rank correlation of the best 10% of individuals and error in predictions. Bias was tested by regression of simulated on estimated breeding values.

**Results:**

The accuracy obtained from the best case scenario was 0.94. Six research groups submitted 19 sets of estimated breeding values. Methods that assumed the same variance for markers showed accuracies, measured as correlations between estimated and simulated values, ranging from 0.75 to 0.89 and rank correlations between 0.58 and 0.70. Methods that allowed different marker variances showed accuracies ranging from 0.86 to 0.94 and rank correlations between 0.69 and 0.82. Methods assuming equal marker variances were generally more biased and showed larger prediction errors.

**Conclusions:**

The best performing methods achieved very high accuracies, close to accuracies achieved in a best case scenario where QTL genotypes were known without error. Methods that allowed different marker variances generally outperformed methods that assumed equal marker variances. Genomic selection methods performed well compared to traditional, pedigree only, methods; all methods showed higher accuracies than those obtained for breeding values estimated solely on pedigree relationships.

## Background

When methods for selection based on many markers across the genome, or genomic selection, were first described [1] the application of genetic marker data in plant and animal breeding programs was still limited [2]. In subsequent years the use of individual markers in breeding programs has increased [3,4]. With the availability of assays that provide genotypes for 50,000 or more markers for each individual, the application of genomic selection has started to take hold in recent years. Especially in dairy cattle the use of genomic selection is becoming common practice [5,6]. In other species the application of genomic selection is being considered or evaluated [7-9].

Methods to deal with these large number of markers in breeding programs were first proposed by [1] after which a number of alternatives have been suggested; e.g. [10,11]. Most methods have been evaluated in simulations and sometimes on real data. Analyses applying genomic BLUP methodology as defined by Meuwissen *et al.*[1], or applying Ridge Regression (RR) [12], assume the same variance for each marker. A series of well-known methods are those named BayesA, BayesB, etc. BayesA assumes the same a priori variance for all markers, where effects are drawn from one distribution [1]. BayesB divides the markers in 2 groups: one group that contributes to the genetic variance and have the same a priori non-zero variance, and another group whose effect are supposed to be zero [1]. Another variant, sometimes referred to as “BayesC”, considers two distributions: one with large effect (that are assumed to be linked to a QTL) and one with small effects (that are assumed to be not linked to a QTL) [11,13].

The organizers of the previous QTL-MAS workshop initiated a comparison of methods using a simulated dataset which resembled a population one might encounter in litter-bearing animals. They concluded that models that include markers as fixed effects were unlikely to provide any gain from the use of markers, while random effects models and especially the Bayesian analyses were most promising [14].

We aimed to compare methods that estimate genomic breeding values (GEBV) in a dataset that one might encounter in both plant and animal breeding programs and added the complexity of repeated measures over time. Participants of the current QTL-MAS workshop 2009 were invited to predict GEBV and describe their methods and results. Predicted GEBV from the different methods were submitted to the QTL-MAS workshop and compared to the simulated or true breeding values (TBV).

## Methods

### Simulated data

18 QTL were simulated affecting a trait called yield that followed a logistic growth curve. The growth curve was determined by 3 parameters and for each parameter, 6 QTL determined the genetic value with one large QTL (50% of genetic variance) and 5 smaller QTL. Phenotypic values for the parameters were simulated with a heritability of 0.50. Workshop participants were provided with genotypes for a set of biallelic markers that did not include the genotypes for the 18 QTL. Data available to participants of QTLMAS XIII consisted of 100 full-sib families which resulted from factorial mating of 20 female and 5 male parents. Each full-sib family consisted of 20 offspring. Parent-offspring relationships were provided, but relationships between parents were not. All offspring had genotypes for 453 markers, distributed over 5 chromosomes of 1 Morgan each. Phenotypes were provided for the offspring of 50 full-sib families and consisted of cumulative yield values at 5 different points in time, the last time point being 530. Further details of the simulation are described elsewhere [15] and the dataset is available from http://www.qtlmas2009.wur.nl/UK/Dataset.

### Best-case analysis

The workshop organizers applied a "best-case" analysis to the simulated data to provide an upper bound of the expected accuracies of the contributed analyses. This best-case analysis made use of additional information which was not provided to the workshop participants. The correct model, a logistic growth curve, was used to estimate 3 growth curve parameters from phenotypes for each individual. More importantly, the true genotypes of the QTL were used. Workshop participants could apply the correct growth model without knowing this was the case, but the actual genotypes could not be used. In the best-case analysis, the true QTL genotypes were used as the only variables in a multitrait fixed regression model to estimate the QTL effects on the 3 growth curve parameters. The estimated growth curve QTL effects were subsequently used to predict breeding value for yield on time point 600 for the unphenotyped individuals.

### Prediction strategies

QTLMAS XIII participants were asked to predict breeding values for the unphenotyped offspring (n = 1000) at time point 600. Timepoint 600 was outside the range of time points for which phenotypes were provided. Several strategies could be followed: 1) predict phenotypes at time point 600, using any of several methods, and use these to predict breeding values, 2) use a function to describe the observed phenotypes, predict breeding values for the parameters of this function and use those to calculate EBV at time point 600, 3) predict breeding values for the 5 different time points and use these to extrapolate to time point 600, using any of several methods.

### Comparison of predicted breeding values

Accuracies of GEBV, reported by workshop participants on unphenotyped offspring, were calculated as the correlation between the GEBV and the TBV. Bias was assessed from the regression of TBV on the GEBV. The ability of methods to identify the best individuals was assessed from the rank correlations of predicted and TBV of individuals in the top 10% of TBV. Mean squared prediction error was calculated after predicted and TBV were centered on zero. The variances of GEBV were calculated and reported as a proportion of the variance of TBV.

## Results

### Best-case analysis

The regression model applied directly to the QTL genotypes resulted in the highest accuracy (0.985) and rank correlation (0.935) of GEBV with TBV of unphenotyped individuals. However, regression of TBV on GEBV resulted in a regression coefficient of 0.847, relatively far away from 1 compared to other methods, The variance of GEBV was also higher (34.3) than the variance of the TBV (25.3) and higher than the variance from any other estimation method.

### Prediction strategies

All authors applied a procedure with two or three steps. In most cases two steps were used with one step to predict phenotypes or breeding values at time point 600 and one step to estimate genetic effects for the markers. The order of these two steps varied and various methods were applied for both extrapolation to time point 600 as well as for the estimation of marker effects.

#### Two step strategies

Three authors [16-18] started with predicting phenotypes on time point 600 which were then used to predict breeding values. Phenotypes were predicted using a Logistic model (model 5) or a Gompertz model (models 1 to 4). Predicted phenotypes at time point 600 were subsequently used in single trait analyses to predict EBV at time point 600.

Three authors [19-21] started by predicting EBV in single trait analyses applied to each of the time points at which phenotypes were available and subsequently used those EBV to extrapolate to an EBV at time point 600. Extrapolation to time point 600 was done using quadratic regression (model 6) or linear regression (models 7 to 18). Linear regression models only used the last 3 time points.

#### Three step strategy

One author [17] (model 19) applied a three step strategy where first a growth model was used, then a model to estimate marker effects and finally again a growth model to predict EBV at time point 600. In step 1 the parameters of a Gompertz model were estimated for each of the individuals with phenotypes. Marker effects were estimated for each of the 3 parameters in the Gompertz model in the second step. The third step used these estimated SNP effects to calculate the EBV of individuals at time point 600.

### Comparison of predicted breeding values

A number of different estimation methods were applied by participants (Table 1). Apart from the prior knowledge analysis performed by the organizers, no fixed effect models were applied to obtain GEBV. One participant [19] applied a pedigree BLUP model (model 8) as one of their approaches which ignored all marker data. We termed 2 models as being a “genomic BLUP” implementation, which meant that each marker was assumed to have the same variance that was not (re-)estimated in the model. The other 16 models were termed “Bayes” models, which meant that they considered a priori assumptions for the marker variances, and estimated the marker variances conditional on the a priori assumptions and the estimated marker effects. All implementations differed in some aspect. The two genomic BLUP implementations used either a genomic relationship matrix [20] (model 6) or a Ridge Regression approach [18] (model 5) both of which are equivalent to a genomic BLUP implementation [22,23]. It needs to be noted that model 6 did not use all available marker information but limited itself to a single chromosome. The 16 models classified as Bayes applied various forms of marker selection or separating markers into groups with different expected effect sizes. Only one group [19] applied the use of haplotype information in some of their models using either identity by descent matrices based on 2 locus haplotypes (model 9) or identity by state haplotypes (models 10 and 11).

**Table 1:** Extrapolation, prediction and estimation methods

			**Step 1**	**Step 2**
**Method**	**Paper**	**Model**	**Extrapolation**	**Prediction**
1	Cleveland	Bayes-A	Gompertz	Bayes-A
2	Cleveland	Lasso	Gompertz	Bayes-Lasso
3	Cleveland	Student- t	Gompertz	Bayes-Student-t
4	Pong-Wong	GEBV2	Gompertz	Bayes-B
5	Schulz-Streeck	Ridge	Logistic	BLUP \ RR
				
			**Step 1**	**Step 2**
**Method**	**Paper**	**Model**	**Prediction**	**Extrapolation**

6	Mucha	SNPL	GRM	quadratic 5 points
7	Veerkamp	14.SNP	Bayes-C	linear 3 points
8	Veerkamp	BLUP	A matrix	linear 3 points
9	Veerkamp	IBD	Bayes-C	linear 3 points
10	Veerkamp	IBS2	Bayes-C	linear 3 points
11	Veerkamp	IBS5	Bayes-C	linear 3 points
12	Veerkamp	SNP1	Bayes-A	linear 3 points
13	Veerkamp	SNP2	Bayes-C	linear 3 points
14	Veerkamp	SNP3	Bayes-C (3dist)	linear 3 points
15	Verbyla	Bayes.BLUP	Bayes	linear 3 points
16	Verbyla	BayesA	Bayes-A	linear 3 points
17	Verbyla	BayesA.B	Bayes	linear 3 points
18	Verbyla	BayesC	Bayes-C	linear 3 points
					
			**Step 1**	**Step 2**	**Step 3**
**Method**	**Paper**	**Model**	**Estimation**	**Prediction**	**Extrapolation**

19	Pong-Wong	GEBV1	Gompertz	Bayes-B	Gompertz
20	QTLMAS	Best case	Logistic	Regression	Logistic
					

#### Accuracy

**Figure 1:**
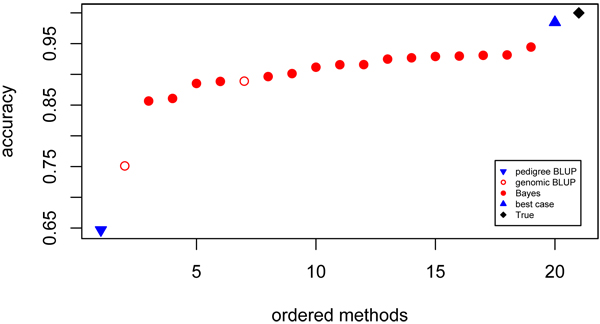
**Accuracy of contributed GEBV** Ordered values for accuracy of predicted breeding values for phenotype at time point 600. Prediction using pedigree (▼), genomic BLUP (○), Bayes (●), best case (▲) models and true breeding values (♦).

The lowest accuracy was 0.647, obtained with pedigree BLUP (model 8), clearly below the second lowest model (model 6) with an accuracy of 0.751. All other methods performed markedly better than the genomic BLUP model 6 (Figure 1). The other genomic BLUP model (model 5) showed an accuracy of 0.889 which was within the lower half of the range of the Bayes models. Accuracies obtained with the Bayes models were between 0.857 to 0.945. The model with the most accurate predictions (model 3) had an accuracy just 4 percent below the results of the best-case analysis.

#### Rank correlations

**Figure 2:**
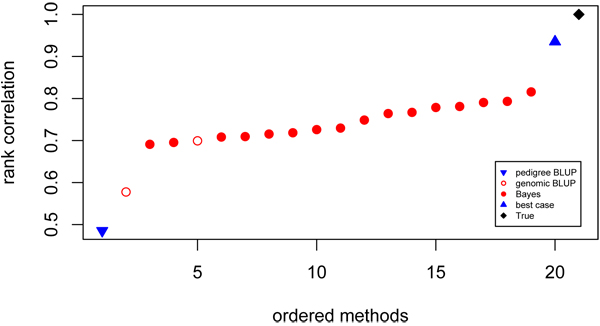
**Rank correlations of contributed GEBV** Ordered values for rank correlations between the predicted and true breeding values of the top 100 TBV individuals. Prediction using pedigree (▼), genomic BLUP (○), Bayes (●), best case (▲) models and true breeding values (♦).

Rank correlations were calculated from the ranking of the top 10% of individuals, based on TBV. The range of rank correlations for the Bayes methods was 0.691 to 0.816 (Figure 2). The two best methods switched positions when measured on rank correlation versus accuracy but overall the evaluation of methods based on accuracy was very similar to evaluation based on their ability to rank the top individuals (correlation = 0.91).

#### Bias

**Figure 3:**
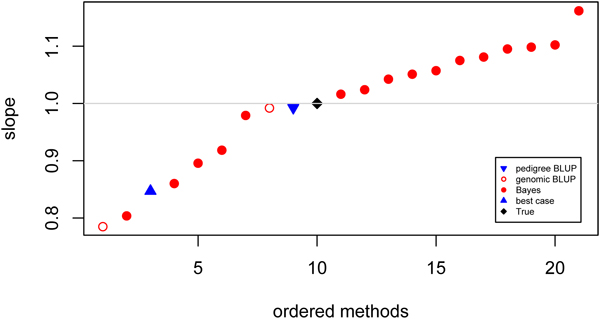
**Bias of contributed GEBV** Ordered values for regression of simulated on predicted breeding values. Prediction using pedigree (▼), genomic BLUP (○), Bayes (●), best case (▲) models and true breeding values (♦).

**Figure 4:**
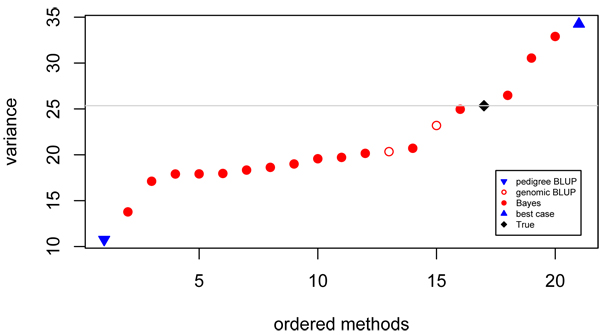
**Variance of contributed GEBV** Ordered values for variance of predicted breeding values. Prediction using pedigree (▼), genomic BLUP (○), Bayes (●), best case (▲) models and true breeding values (♦).

The pedigree BLUP analysis was found to have a regression coefficient that was closest to 1 (Figure 3). The genomic BLUP method by Schulz-Streek 5 also yielded a regression coefficient very close to 1, while the variance of GEBV from that model was approximately twice the variance of EBV obtained with pedigree BLUP (Table 2, Figure 4). Regression coefficients of Bayes models ranged from 0.804 for a Bayesian implementation of LASSO (model 2) to 1.16 for a BayesB implementation (model 16).

**Table 2:** Results from comparison to simulated breeding values (TBV)

**Analysis**	**Method**	**Var**	**Acc**	**MSEP**	**Rank**	**Regr**
0	TBV	25.346	1	0	1	1
1	Cle_Bayes.A	26.490	0.916	4.369	0.749	0.896
2	Cle_Lasso	32.909	0.916	5.337	0.716	0.804
3	Cle_Student.t	30.554	0.945	3.322	0.791	0.860
4	Pon_GEBV2^1^	18.638	0.901	4.794	0.726	1.051
5	Sch_EBV600	20.354	0.889	5.303	0.700	0.992
6	Muc_SNPL	23.204	0.751	12.101	0.578	0.785
7	Vee_14.SNP	20.166	0.930	3.460	0.764	1.043
8	Vee_BLUP	10.763	0.647	14.728	0.485	0.993
9	Vee_IBD	19.009	0.931	3.475	0.767	1.075
10	Vee_IBS2	18.344	0.932	3.504	0.816	1.095
11	Vee_IBS5	19.579	0.929	3.517	0.781	1.057
12	Vee_SNP1	24.975	0.912	4.433	0.719	0.919
13	Vee_SNP2	17.974	0.925	3.828	0.793	1.098
14	Vee_SNP3	17.928	0.927	3.744	0.779	1.102
15	Ver_Bayes.BLUP	20.721	0.885	5.479	0.691	0.979
16	Ver_BayesA	13.783	0.857	7.092	0.696	1.162
17	Ver_BayesA.B	17.124	0.889	5.435	0.730	1.081
18	Ver_BayesC	17.914	0.861	6.561	0.710	1.024
19	Pon_GEBV1	19.726	0.897	4.971	0.709	1.016
20	best_case	34.256	0.985	1.572	0.935	0.847

^1^After the QTLMAS workshop an error in the implemented software was found by the participant. The adjusted implementation yielded an accuracy of 0.947.

#### Prediction error

**Figure 5:**
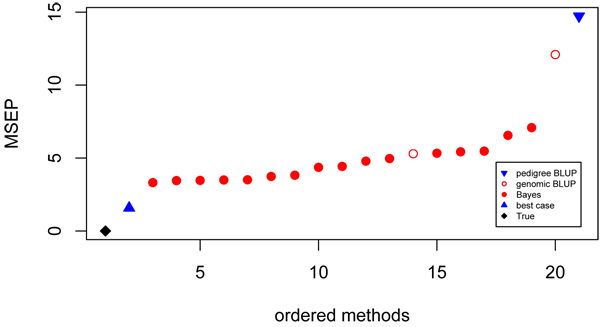
**Prediction error of contributed GEBV** Ordered values for mean squared prediction error of predicted breeding values. Prediction using pedigree (▼), genomic BLUP (○), Bayes (●), best case (▲) models and true breeding values (♦).

Average prediction error was largest for the pedigree BLUP EBV, which is due to the relatively low accuracies for this method and hence significant shrinkage of the resulting EBV (Figure 5). Smallest prediction errors were obtained with the most accurate prediction method (model 3). Average prediction error showed a very strong correlation with accuracy (r = -0.99).

## Discussion

### 2-step and 3-step methods

Accuracy of GEBV from the 3-step method (model 19) was 0.897, which was very similar to the average accuracy, 0.893, of all the other Bayes methods. Because the simulated QTL affect the parameters of the true logistic growth curve it might have been expected that methods that look for associations of markers with these underlying parameters had an advantage. In this dataset this does however not appear to be the case. It was found that extrapolation to time point 600 was not a big challenge because for most individuals this time point was within the part of the growth curve where growth was almost linear. In fact, two authors extrapolated exactly this way, by linear regression on the last three time-points and obtained high accuracies. The impact of methods for extrapolation would have been bigger when a time-point closer to the asymptote would have been chosen. However, extrapolation of the data was a secondary objective of the QTLMAS workshop whereas comparison of methods to obtain GEBV was a primary objective.

### Bayes and genomic BLUP methods

QTL contributing to the three parameters of the growth curve were unequal in size. This was expected to favor Bayes methods over genomic BLUP methods that assume the same variance for all markers. This difference was not directly apparent from the results presented in this comparison as one of the genomic BLUP models (model 6) resulted in a lower accuracy (0.751) compared to all other marker methods while the accuracy (0.889) from the other genomic BLUP method (model 5) was higher than some of the Bayes methods. The GEBV from model 6 were obtained using a genetic covariance matrix build from a subset of 90 markers that were selected from only the first of the five chromosomes. The resulting GEBV may have reduced accuracies compared to other methods because not all information was used. However, a method which only selected 14 markers (model 7) still gave a high accuracy (0.930). These 14 markers were selected based on a QTL analysis while the 90 markers selected for model 6 were selected solely on the fact that they map to chromosome one.

Most likely the use of a polygenic component increased the accuracy of model 5. While a polygenic component was not used by model 6, some of the Bayes methods did include polygenes (models 7 to 18). In addition to the relatively high accuracy, the best genomic BLUP model (model 5) produced unbiased GEBV where many of the other methods showed moderately to severely biased results.

The structure of the data was such, that full sib families were either completely genotyped, or completely not genotyped. This structure was chosen, to avoid that models would benefit too much from close relationships between the animals in the training and validation data. The larger the distance between training and validation, the more emphasis on LD information to predict GEBV of animals without phenotypes [24]. Bayes methods can employ LD to focus variance on specific parts of the genome, where genomic BLUP methods only employ the genomic relation over the whole genome. Therefore, in addition to the small number of QTL with relatively large effect, most likely the population structure was also more beneficial for the Bayes compared to the genomic BLUP models.

### Number of markers included in the model

The proportion of markers, π, selected into the model or into the distribution of large marker effects was reported by Pong-Wong and found to be relatively high, even close to 1 for one of their methods where they tried to let the model decide on the value of π . Nevertheless, the proportion of markers that was included in the distribution of markers with large effect in more than half of the cycles (i.e. that had a posterior probability > 0.5), was limited. The posterior proportions in the various distributions were not reported by the other authors. Results obtained with a method that solely included 14 markers (model 7) show that very high accuracies could be obtained, at least in this dataset, with a small number, and a small proportion of the total number of markers selected into the model. The 14 SNPs included in model 7 were selected based on their association with the phenotype and analysed with a Bayes model. Preselection of SNPs and analysing them as fixed effects has not been considered by any of the participants. Results from QTLMAS comparison in 2008 [14] as well as the first comparison to genomic BLUP, BayesA and BayesB [1] already showed a low accuracy for these fixed effects models which can be expected when many markers are available to be selected into the model.

## Conclusions

Accuracies of GEBV were always higher than those estimated based on pedigree alone (model 8). Methods that allow different variances of markers generally performed better than genomic BLUP methods that assume equal variance for all markers but differences were not very large, except when only a portion of the genome was used (model 6). The best Bayes method achieved an accuracy that was 0.056 higher than the best genomic BLUP method. The simulated QTL varied strongly in size which will have favored the Bayes methods in the comparison of accuracies between the two types of methods. The highest accuracies obtained were very close to those from the best case analysis, where knowledge about QTL genotypes was used. Methods to extrapolate to time point 600 from the observed phenotypes at time points up to 530 appear to have had a minor impact on the accuracies of GEBV.

## Competing interests

The authors declare that they have no competing interests.

## Authors' contributions

JB performed the comparative analyses, MC performed the best-case analysis, JB and MC drafted the paper. All authors contributed to the design of the study and the methods for comparative analyses. CM, MB and AC contributed to discussion of the results and revision of the paper.
